# Increased mortality among men aged 50 years old or above with elevated IgA anti-transglutaminase antibodies: NHANES III

**DOI:** 10.1186/s12876-016-0547-8

**Published:** 2016-11-03

**Authors:** Alberto Rubio-Tapia, Jonas F. Ludvigsson, Rok Seon Choung, Tricia L. Brantner, S. Vincent Rajkumar, Ola Landgren, Joseph A. Murray

**Affiliations:** 1 Division of Gastroenterology and Hepatology, Department of Medicine, Mayo Clinic, Rochester, MN 55905 USA; 2 Deparment of Pediatrics, Orebro University Hospital, Orebro, Sweden; 3 Department of Medical Epidemiology and Biostatistics, Karolinska Institutet, Stockholm, Sweden; 4 Division of Hematology, Department of Medicine, Mayo Clinic, Rochester, MN USA; 5 Multiple Myeloma Section, Lymphoid Malignancy Branch, National Cancer Institute, National Institutes of Health, Bethesda, MD USA; 6 Myeloma Service, Department of Medicine, Memorial Sloan Kettering Cancer Center, New York, NY USA; 7 National Institute of Diabetes and Digestive and Kidney Diseases, National Institutes of Health, Department of Health and Human Services, Bethesda, MD USA

**Keywords:** Epidemiology, Survival, Transglutaminase

## Abstract

**Background:**

Immunoglobulin A (IgA) antibodies to tissue transglutaminase (tTG) are the serologic test of choice for diagnosing celiac disease (CD). Our aim was to determine if elevated IgA anti-tTG were associated with increased mortality risk.

**Methods:**

Stored serum samples of National Health and Nutrition Examination Survey (NHANES) III (1988–1992) were available for 6032 individuals aged 50 years old or above, which were screened for IgA anti-tTG, and if positive, for IgA endomysial antibodies. Mortality was determined from the National Death Index records through 2006. Hazard ratios were calculated through Cox proportional hazards regression.

**Results:**

From a total of 6032, 85 participants tested positive for IgA anti-tTG (1.4 %) and 5947 tested negative. After a median follow-up of 13 years, IgA anti-tTG positive participants were at increased risk of death in both crude (HR = 1.68; 95 % CI = 1.30–2.18) and adjusted analyses (adjusted hazard ratio = 1.43; 95 % CI = 1.10–1.85) as compared to IgA anti-tTG negative participants. The excess mortality was restricted to IgA anti-tTG positive males (adjusted hazard ratio = 1.69 (95 % CI = 1.26–2.29), as opposed to a hazard ratio of 0.96 (95 % CI = 0.57–1.62) among IgA anti-tTG positive females. Although the most common cause of death in IgA anti-tTG positive participants was cardiovascular disease (36 %), the increased hazard ratio was only observed in respiratory cause of death as compared to IgA anti-tTG negative participants (adjusted hazard ratio = 5.11; 2.76–9.46).

**Conclusion:**

Men aged 50 years old or above participants of NHANES III with elevated IgA anti-tTG antibodies had increased mortality risk. Elevated IgA anti-tTG antibodies could be a nonspecific marker of serious disease in older men.

## Background

Celiac disease (CD) affects almost 1 % of the American population yet most cases remain undiagnosed (~80 % of cases) and the diagnosis is often delayed after patients first present with typical symptoms [1, 2]. Among current available serologic tests for CD, Immunoglobulin A (IgA) antibodies to tissue transglutaminase (tTG) are the serologic test of choice for diagnosing CD [3]. The sensitivity and specificity of IgA anti-tTG antibodies for CD are >95 % each [4]. However, diagnostic performance of IgA anti-tTG antibody tests varies among laboratories due to lack of standardization of the anti-tTG assay [5]. Moreover, these antibodies can be found in patients without CD but having other conditions such as chronic liver disease, heart failure, and autoimmune disorders [6, 7]. Thus, the presence of IgA anti-tTG antibodies is not exclusive for CD especially in the context of negative endomysial antibodies (EMA), the most specific marker for CD [8].

Several population-based studies have shown an association of increased mortality risk in patients with CD [9–12], but other studies have not [13–15]. Moreover, studies on the mortality associated with undiagnosed CD, which was detected by IgA tTG antibodies and/or EMA positivity, have reported more contradictory findings [13, 14, 16–18]. Although, based on the IgA tTG assays, a European study showed excess mortality risk particularly due to cancer among individuals with elevated IgA anti-tTG antibodies over 10 years’ follow-up period [18], no such study has been performed in a nationwide sample of the United States with systematical approaches.

Our main aim was to determine whether elevated IgA anti-tTG antibodies were associated with higher mortality risk in a representative sample of the United States population aged 50 years or older.

## Methods

### Subjects

The National Health and Nutrition Survey (NHANES) is conducted by the National Center for Health Statistics of the Center for Disease Control and Prevention. It collates nationally representative data on the health and nutritional status of the non-institutionalized, civilian population of the United States. It utilizes a complex, stratified, and multistage probability sampling design and collects information from participants using standardized household interviews, physical examinations, and testing of biologic samples. More detailed information on the survey design for the NHANES, including approval from the institutional review board for data collection and analysis, is available from the survey documentation [1, 19]. For our study, the NHANES III (1988–1994) database and samples were used.

### Laboratory methods to detect CD

Stored serum samples of participants aged 50 years or above from NHANES III were shipped to the Celiac Disease Research Laboratory at Mayo Clinic, Rochester, MN, USA, which performed serological testing using laboratory techniques that have been described previously [1]. Briefly, serum samples were tested for IgA anti-tTG antibodies using an enzyme-linked immunosorbent assay for the semiquantitative detection of IgA antibodies to tTG (CD autoantigen) in human serum using human recombinant antigen (Inova; San Diego, CA). Test results were considered negative if <4.0 U/mL; weakly positive if 4–10 U/mL; and positive if >10 U/mL. Serum samples with abnormal IgA-tTG antibodies results (titers ≥4.0 U/mL) were tested for IgA-EMA by indirect immunofluorescence using the reticulin component of the endomysium of the smooth muscle in monkey esophagus tissue (Inova; San Diego, CA). The result was considered positive if fluorescence was seen at a dilution of ≥1:5 as previously reported by our group and many others.

### Definition of case and referent subjects

For analytic purposes, participants with abnormal IgA anti-tTG antibodies were considered cases and participants with normal IgA anti-tTG antibodies were considered referent (no exposed) subjects.

### Follow-up procedures and ascertainment of death

We used the National Center for Health Statistics updated mortality linkage of the Third National Health and Nutrition Examination Survey (NHANES III) to death certificate data found in the National Death Index. The updated NHANES III Linked Mortality File provides mortality follow-up data from the date of NHANES III survey participation (1988–1994) through December 31, 2006. Mortality ascertainment is based upon the results from a probabilistic match between NHANES III and National Death Index death certificate records [20].

### Statistical analysis

For the univariate analysis we compared proportions using the Chi-square test, and medians using the Mann-Whitney U-test. Hazard ratios (HRs) were calculated through Cox regression. We performed crude analyses, as well as analyses adjusted for age (continuous variable), sex, ever-smoking (yes = defined as having smoked >100 cigarettes (5 packs) during lifetime), and race (non-Hispanic white, non-Hispanic black, Hispanic, and other). The proportional hazards assumption was not violated as shown by log-minus-log curves. Still, given the different HR especially in the first year of follow-up vs. later we calculated HRs for death according to follow-up (<1 years, 1- < 5 years; and ≥5 years). We also calculated stratified analyses according to sex, age at testing (50–59, 60–69, 70–79, and 80+ years) as well as according to race.

Follow-up began on the date of IgA anti-tTG antibody testing and ended with death or end of study period (Dec 31, 2006) whichever occurred first. We calculated absolute mortality rates according to number of deaths per 1000 person-years. To ensure comparability between IgA anti-tTG + and IgA anti-tTG - groups we estimated an age- and sex-standardized mortality in IgA anti-tTG - controls as the mortality rate in IgA anti-tTG + individuals divided by the adjusted HR for death. Excess mortality was the difference in mortality between IgA positive individuals and IgA anti-tTG negative individuals per 1000 person-years. We used SPSS 20.0 and JMP for all analyses. P-values were two-sided and considered statistically significant if <0.05.

## Results

### Baseline characteristics

Out of 6557 NHANES III participants, a subset of 6034 were tested for IgA-tTG antibodies. Of these, NHANES categorized two individuals as ineligible for further analyses due to missing information on vital status. All further data refer to the remaining 6032 individuals out of whom, 85 tested positive for IgA anti-tTG antibody (1.4 %; 95 % CI: 1.1–1.7 %), out of which 7 individuals also tested positive for endomysial antibodies (EMA). Among 85 positive tTG individuals, 87 % (74/85) were weakly positive to IgA anti-tTG. Two thirds of the IgA anti-tTG antibody positive individuals were males (54/85; 64 %) compared with only 47 % (2793/5947) of IgA anti-TTG negative individuals (the latter hereby called referent individuals). The follow-up was significantly shorter in IgA-tTG antibody positive individuals than in referent individuals, potentially due to a higher rate of death among IgA anti-tTG antibody positive individuals. Table 1 shows the demographic characteristics of study participants according to the status of IgA anti-tTG antibody.

**Table 1:** Characteristics of study participants

Variable	tTGA+ *n* = 85	tTGA- *n* = 5947	P value
Age in years ; median (range)	69 (50–90)	67 (50–90)	0.139
Follow-up in years; median (range)	11 (0–18)	13 (0–18)	0.001
Male (%)	54 (64)	2793 (47)	0.002
Ever smoker^a^ (%)	51 (60)	3211 (54)	0.270
Race/ethnicity (%)			0.589
Non-Hispanic White	41 (48)	3298 (55)	
Non-Hispanic Black	21 (25)	1209 (20)	
Mexican-American	19 (22)	1216 (20)	
Other	4 (5)	224 (4)	

*tTGA* tissue transglutaminase antibody

^a^Smoked at least 100 cigarettes (5 packs) during lifetime

### Mortality

**Fig. 1:**
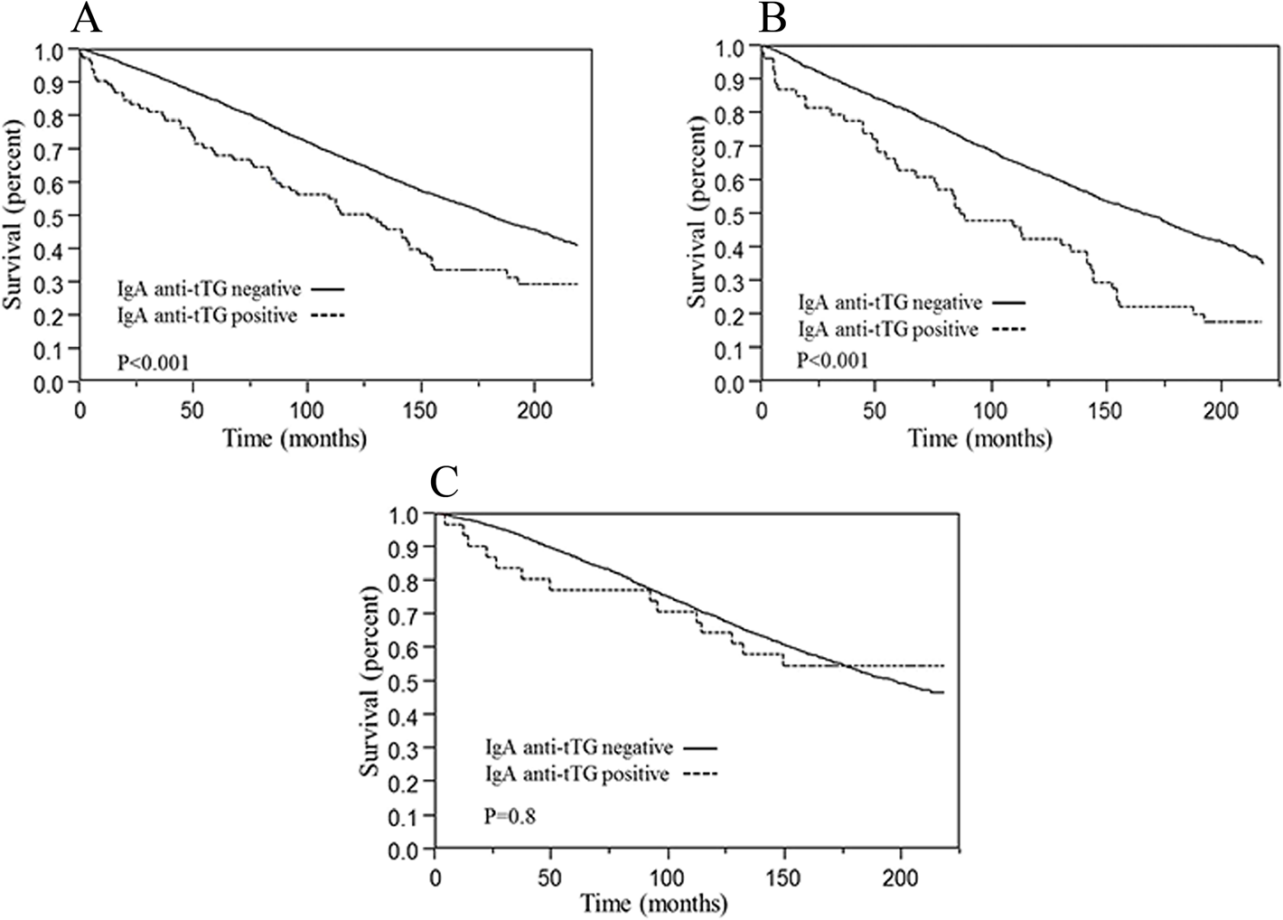
Unadjusted Kaplan-Meier survival curves for all-cause mortality by IgA anti-tTG antibodies status in the National Health and Nutrition Examination Survey (NHANES) III from 1988 to 2006. Overall survival for all subjects (**a**), males (**b**), and females (**c**) with elevated IgA anti-tTG antibodies vs. IgA anti-tTG negative individuals

During follow-up, 58 IgA anti-tTG antibody positive and 2996 referent individuals died (68 vs. 50 %, respectively). The median age at death was 72 years among IgA anti-tTG antibody positive individuals vs. 74 years among referent individuals. Figure 1a shows the survival curves of IgA anti-tTG antibody positive individuals and referent individuals, which suggested better survival in referent individuals (*p* < 0.001). However, when gender effects were evaluated, IgA anti-tTG antibody positive individuals had significantly poor survival only in men (*p* < 0.001) not in women (*p* = 0.8), compared to referent individuals (Fig. 1b and c, respectively). Of the deceased individuals with available data on risk factors (*n* = 3016), 17.2 % of IgA anti-tTG positive individuals (referent: 11.5 %) had diabetes, and 8.6 % (referent: 12.5 %) had hypertension. IgA anti-tTG antibody positive individuals were at increased risk of death in both crude (HR = 1.68; 95 % CI = 1.30–2.18) and adjusted analyses (adjusted for age, sex, smoking, and race, HR = 1.43; 95 % CI = 1.10–1.85) (Table 2). The risk increase was especially high in the first year after testing (HR = 3.52; 95 % CI = 1.72–7.20), and after the first year of follow-up there was only a borderline excess mortality (HR = 1.30; 95 % CI = 0.98–1.72).

**Table 2:** Mortality in IgA anti-tTG positive individuals based on follow-up time^a^

Follow-up	tTGA+ Deaths	tTGA- Deaths	Adjusted HR; 95 % CI	P value	Absolute risk/1000 PYAR	Excess risk/1000 PYAR
All	58	2996	1.43; 1.10–1.85	0.007	74	22
Year <1	8	134	3.52; 1.72–7.20	0.001	100	72
1–4.99	19	778	1.58; 1.00–2.49	0.051	70	26
5+	31	2084	1.18; 0.83–1.69	0.354	71	11

Reference is general population comparator cohort

*tTGA* tissue transglutaminase antibody, *PYAR* Person-years at risk

^a^All hazard ratios (HRs) were adjusted for sex, race, age, and ever smoking. See text for additional explanation

The excess mortality was restricted to IgA anti-tTG antibody positive males where the HR for death was 1.69, as opposed to 0.96 in females. This difference was statistically significant (p for interaction between IgA anti-tTG status and sex was 0.04). In contrast, mortality HRs did not differ by age at IgA anti-tTG antibody testing (p for interaction = 0.97) or by race (p for interaction = 0.21) (Table 3).

**Table 3:** Mortality in IgA anti-tTG positive individuals (subgroup analyses)

Subgroup	tTGA+ Deaths	tTGA- Deaths	Adjusted HR; 95 % CI	P value	Absolute risk/1000 PYAR	Excess risk/1000 PYAR
Sex						
Males	44	1535	1.69; 1.26–2.29	0.001	96	39
Females	14	1461	0.96; 0.57–1.62	0.881	42	−2
Age						
50–59 years	3	272	1.91; 0.61–6.02	0.267	21	10
60–69 years	20	766	1.58; 1.01–2.47	0.044	54	20
70–79 years	22	940	1.49; 0.97–2.27	0.068	103	34
80+ years	13	1018	1.41; 0.81–2.47	0.222	217	63
Race						
Non-Hispanic White	29	1794	1.58; 1.09–2.28	0.015	77	28
Non-Hispanic Black	13	603	1.25; 0.72–2.17	0.424	68	14
Mexican-American	13	532	1.23; 0.71–2.15	0.461	68	13
Other	3	67	4.75; 1.45–15.55	0.010	97	76

Reference is general population comparator cohort

*tTGA* tissue transglutaminase antibody, *PYAR* Person-years at risk

Table 4 shows the number of total and cause-specific deaths in IgA anti-tTG positive individuals. The most common cause of death in IgA anti-tTG positive individuals was cardiovascular disease (36 %), however the relative risk of cardiovascular death was not statistically significant (HR = 1.10), compared to IgA anti-tTG negative individuals. While only 6 % of referent individuals died from respiratory disease, the corresponding fraction was 19 % in IgA anti-tTG antibody positive individuals with a relative risk of more than 5 (HR = 5.11, 95 % CI = 2.76–9.46). However, the median of IgA anti-tTG antibody titers (4.91, interquartile range = 4.38–6.69) in people who died from respiratory cause was similar to that of other groups (5.38, interquartile range = 4.40–6.66) (*p* = 0.5) among IgA anti-tTG positive individuals.

**Table 4:** Cause-specific mortality in IgA anti-tTG positive individuals

Follow-up	tTGA+ Deaths	tTGA- Deaths	Adjusted HR; 95 % CI	P value	Absolute risk/1000 PYAR	Excess risk/1000 PYAR
Cardiovascular	21	1372	1.10; 0.72–1.70	0.653	27	2
Cancer	13	662	1.48; 0.85–2.56	0.164	16	5
Respiratory	11	180	5.11; 2.76–9.46	<0.001	14	11
Other	13	743	1.30; 0.75–2.25	0.351	16	4

Reference is general population comparator cohort

*tTGA* tissue transglutaminase antibody, *PYAR* Person-years at risk

## Discussion

By testing for IgA anti-tTG antibody stored sera drawn from a nationally representative sample of individuals aged 50 or more, we were able to estimate mortality risk over time associated with elevated IgA anti-tTG antibody as compared to no exposed individuals in the United States population. This study yielded three major findings. First, the presence of elevated IgA anti-tTG antibodies was associated with an increased mortality risk, especially in respiratory cause of death. This finding is consistent with a prior report from the KORA/MONICA Augsburg cohort study [18]. Second, mortality risk was limited to men. Third, excess mortality risk is unlikely to be related to undiagnosed CD as just a minority had double positive serology (IgA anti-tTG positive and IgA EMA). We can speculate that elevated IgA anti-tTG antibodies could be a marker of serious disease in older men such as chronic pulmonary diseases.

Some studies showed an increased risk of mortality in undiagnosed CD patients compared to controls [10, 17]. Especially, Metzger et al. [18] showed that individuals with elevated IgA anti-tTG antibodies present with an excess of all-cause mortality, particularly due to cancer. In addition, we found mortality risk was significantly higher the year after testing for IgA anti-tTG antibodies. Thus, it is conceivable that presence of IgA anti-tTG antibodies is associated with serious disease. This is in contrast with decades needed to demonstrate excess mortality in subjects with undiagnosed CD (defined by double positive serology including both IgA anti-tTG antibodies and IgA EMA) [17]. Although excess of overall and respiratory mortality have been demonstrated in CD [10], it is unlikely that undiagnosed CD fully explain our findings as just a minority (8 %) of IgA anti-tTG antibody subjects had double positive serology. We lacked data on small intestinal histopathology and biopsies were not used to confirm (or rule out) the presence of CD in our study population.

We observed an increased risk of respiratory deaths among those with elevated IgA anti-tTG antibodies. This findings is similar to a previous study from Sweden [10] that reported an excess respiratory mortality in children with latent CD (HR = 2.83; 95 % CI = 1.41–5.67), who had normal mucosal with positive CD serology. Furthermore, older reports also showed the association of lung disease and CD [21, 22]. Robinsons et al. [21] showed that the higher occurrence of CD in patients with farmer’s lung, and Axmacher et al. [22] also described an increased occurrence of CD in Farmers’ lung. Interestingly, the majority of cases (about three fourths) were men in these studies. Our study also showed poor survival in IgA anti-tTG antibody positive individuals, especially in men. This finding suggested that there is a possible association between elevated IgA anti-tTG antibody and respiratory disease, but the role of undiagnosed CD to explain excess respiratory disease mortality remains unclear. Moreover, we did not find any association between higher titers of tTG-IgA and respiratory cause of death. Our study also showed no statistically significant increased risk of cardiovascular death or malignancy related death in IgA anti-tTG antibodies individuals. In contrast, Metzger et al. [18] reported an increased hazard ratio of cancer mortality (HR = 3.62) for individuals with an elevated IgA anti-tTG concentration relative to those with a lower concentration. However, based on a small number of cancer mortality cases, their results were inconclusive.

Our study was limited by several factors. Mortality data was obtained through the National Death Index and the cause of death reported on death certificates may suffer from inaccuracies or misclassification. Also, we note a small sample size in subjects with IgA anti-tTG positive and EMA (*n* = 7) to provide robust estimates for mortality of CD autoimmunity. However, NHANES is the unique database that allows for an unbiased study of the sero-epidemiology related to CD in the US civilian population, since NHANES III is a population-based survey, representative of the US population. Another strength is quite a long follow-up period for estimating mortality rates in the IgA anti-tTG positive subgroup. Moreover, this study applied a highly sensitive and specific serologic test to a relatively large number of subjects from the general US population. Testing was performed by the same laboratory technicians with advanced training in CD using the same methods and reagents, thus limiting biases due to personnel or differences due to specific reagents.

## Conclusions

In conclusion, men aged 50 years old or above participants of NHANES III with elevated IgA anti-tTG antibodies had increased mortality risk. Mortality risk is not related to undiagnosed CD as just a minority had double positive serology (IgA anti-tTG positive and IgA EMA). Elevated IgA anti-tTG antibodies could be a nonspecific marker of serious disease in older men.

## Abbreviations

CD: Celiac disease
EMA: Endomysial antibodies
HR: Hazard ratio
IgA: Immunoglobulin A
NHANES: National Health and Nutrition Examination Survey
tTG: Tissue transglutaminase
